# Implementation of an efficient SARS-CoV-2 specimen pooling strategy for high throughput diagnostic testing

**DOI:** 10.1038/s41598-021-96934-z

**Published:** 2021-09-07

**Authors:** Lavanya Singh, Ugochukwu J. Anyaneji, Wilfred Ndifon, Neil Turok, Stacey A. Mattison, Richard Lessells, Ilya Sinayskiy, Emmanuel J. San, Houriiyah Tegally, Shaun Barnett, Trevor Lorimer, Francesco Petruccione, Tulio de Oliveira

**Affiliations:** 1 KwaZulu-Natal Research Innovation and Sequencing Platform (KRISP), School of Laboratory Medicine & Medical Sciences, University of KwaZulu-Natal, Durban, South Africa; 2 African Institute for Mathematical Sciences, The Next Einstein Initiative, Kigali, Rwanda; 3 Higgs Centre for Theoretical Physics, School of Physics and Astronomy, University of Edinburgh, Edinburgh, UK; 4 School of Chemistry and Physics, University of Kwa-Zulu Natal, Westville, South Africa; 5 National Institute for Theoretical and Computational Sciences (NITheCS), KwaZulu-Natal, South Africa; 6 Discipline of Electrical, Electronic and Computer Engineering, University of KwaZulu-Natal, Durban, South Africa

**Keywords:** Infectious-disease diagnostics, SARS-CoV-2

## Abstract

The rapid identification and isolation of infected individuals remains a key strategy for controlling the spread of SARS-CoV-2. Frequent testing of populations to detect infection early in asymptomatic or presymptomatic individuals can be a powerful tool for intercepting transmission, especially when the viral prevalence is low. However, RT-PCR testing—the gold standard of SARS-CoV-2 diagnosis—is expensive, making regular testing of every individual unfeasible. Sample pooling is one approach to lowering costs. By combining samples and testing them in groups the number of tests required is reduced, substantially lowering costs. Here we report on the implementation of pooling strategies using 3-d and 4-d hypercubes to test a professional sports team in South Africa. We have shown that infected samples can be reliably detected in groups of 27 and 81, with minimal loss of assay sensitivity for samples with individual Ct values of up to 32. We report on the automation of sample pooling, using a liquid-handling robot and an automated web interface to identify positive samples. We conclude that hypercube pooling allows for the reliable RT-PCR detection of SARS-CoV-2 infection, at significantly lower costs than lateral flow antigen (LFA) tests.

## Introduction

A novel coronavirus, SARS-CoV-2, emerged at the end of 2019 in the city of Wuhan, China. The highly transmissible nature of SARS-CoV-2^1^ has resulted in a pandemic which continues to persist. South Africa (SA) and other African countries are currently facing a resurgence or “third wave” of infections, which in some countries is more severe than previously experienced^2–4^. Although there are approved vaccines against SARS-CoV-2, these are not yet available in amounts sufficient to control the pandemic. To make matters worse, there are new SARS-CoV-2 variants (which are typically identified using genomics surveillance tools^5,6^) that have been identified in SA^7^, which may be associated with higher transmissibility and hence a more rapid spread of the virus. There is therefore an urgent need for more efficient population screening and the isolation of infected individuals to reduce the transmission of SARS-CoV-2.

Presymptomatic or asymptomatic individuals, who are infectious viral carriers^8^, are the hidden drivers of the pandemic. They represent an estimated proportion ranging from 18 to 81% of infections^9^, thereby posing a major challenge to the containment of SARS-CoV-2. If such individuals can be efficiently detected through frequent, repeated population testing at scale and thereby enabled to isolate before they infect others, the spread of the virus can be prevented^8^. Therefore, efficient and affordable, high throughput SARS-CoV-2 testing is highly desirable as a means of controlling the pandemic.

Reverse-transcription real-time polymerase chain reaction (RT-PCR) testing is the gold-standard technology used for SARS-CoV-2 diagnosis. This test can cost up to US$ 56 (ZAR 850) per test, making high-throughput RT-PCR testing of every individual impractical. Sample pooling offers an attractive solution. By combining samples and testing them together, instead of performing individual tests, one can significantly reduce the number of tests and the associated labour and consumable costs. This method was first proposed by Dorfman^10^ in 1943. Hypercube pooling, developed by Mutesa et al.^11^, requires even fewer tests and yields greater cost savings. For example, at viral prevalences *p* < 0.05%, hypercube pooling yields a 100-fold cost-reduction as opposed to a 22-fold for Dorfman’s algorithm. Hypercube pooling therefore offers a highly affordable means of testing large numbers of samples. We also describe a quantitative cost comparison with lateral flow antigen (LFA) tests, finding hypercube pooling to have a significant cost advantage at low viral prevalence (Supplementary Information—Cost comparison of hypercube-based pooled tests vs. lateral flow antigen tests**)**.

**Figure 1:**
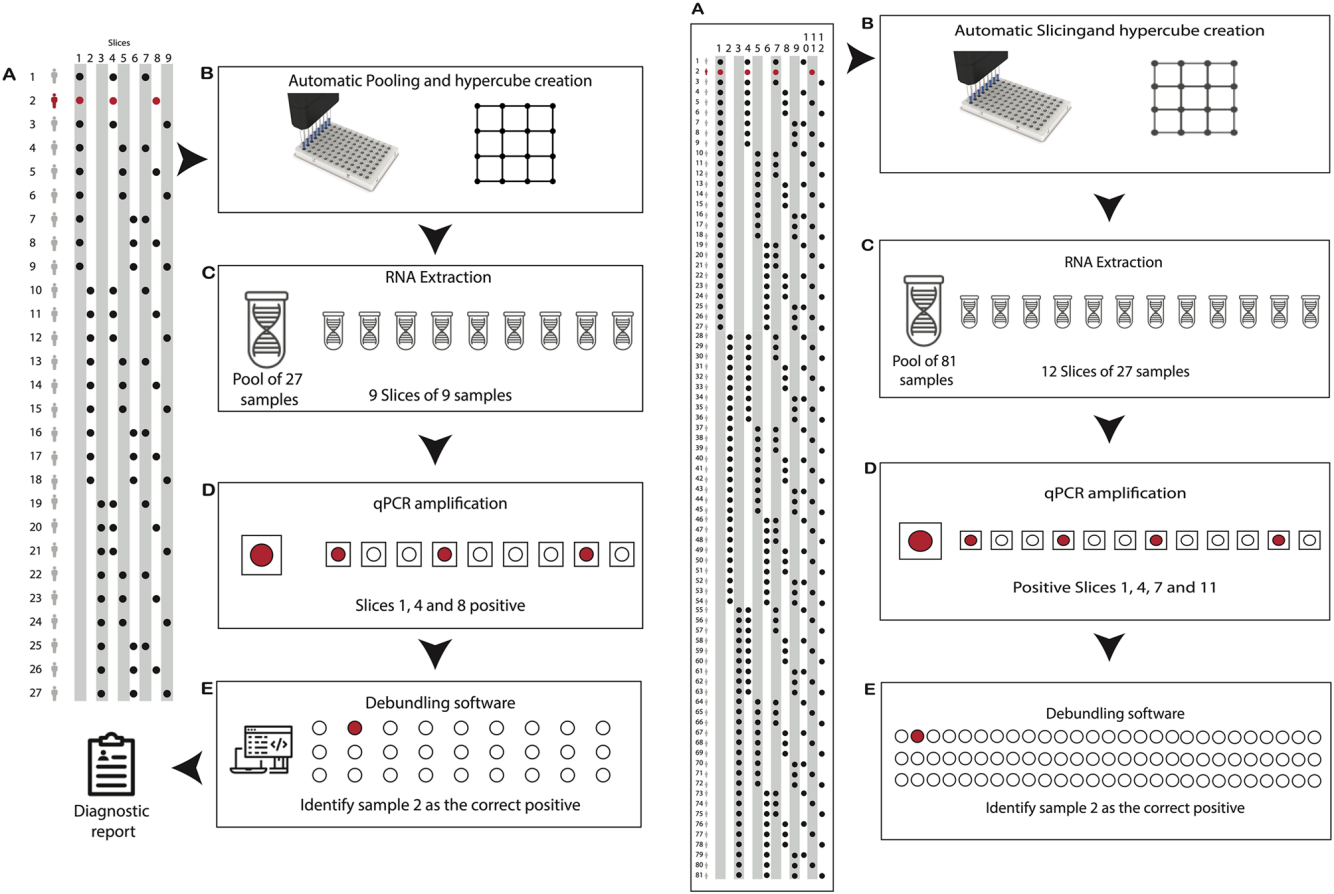
Examples of 3-dimensional (left) and 4-dimensional (right) hypercube workflows; (**A**) one positive sample (red, sample 2) is contained in a group of 27 samples or, respectively, 81 samples; (**B**) 9 (resp. 12) slice pools, each consisting of 9 (resp. 27) sub-samples, are created using a liquid handling robot; (**C**) RNA extraction is done on the group pool of 27 (resp. 81) samples (1 reaction) and 9 (resp. 12) slice pools (9 or 12 reactions); (**D**) qPCR results are positive for the group of 27 (resp. 81) samples and the respective slices 1, 4 and 8 (resp. 1, 4, 7, and 11); (**E**) De-bundling software is used for the automatic determination of the positive sample in the hypercube and for generating individual diagnostic reports.

Hypercube pooling has been eloquently explained in the literature^11^. Briefly, the samples to be tested are divided into equally sized subsamples which are pooled together according to a mathematical algorithm. Here we report on pooled testing methods which can uniquely identify infected samples among groups of 27 and 81 samples (using 3- and 4-dimensional hypercubes, respectively), in far fewer than 27 or 81 tests. If the group is negative, then all individual samples in that group are deemed negative. If a group is positive, then its sub-samples are recombined in the form of slices within the hypercube corresponding to different overlapping sub-pools. Groups of 27 or 81 samples are sub-pooled into 9 and 12 slices respectively, each consisting of 9 or 27 subsamples respectively. Each individual sample is then represented in 3 or 4 different slices respectively, and the test results for the slices can be used to infer which sample is positive, based on its consistent detection within each of the hypercube slices, without an individual test ever being required. The slicing patterns for these groups are shown in the Supplementary Information Tables S1 and S2 and the complete workflows are presented in Fig. 1.

Although the pooling strategy described above offers considerable benefits over individual testing, it does involve tedious and repetitive pipetting steps, which can be cumbersome when performed manually and can raise the risk of human error. For example, a 3-d hypercube involves 108 precise pipetting steps in different combinations, i.e., a group-level pool of 27 sub-samples plus 9 slice-level sub-pools of 9 sub-samples each. The 4-d hypercube involves 405 pipetting steps, i.e., the first group-level pool of 81 sub-samples plus 12 slice-level sub-pools of 27 sub-samples each. To reduce these intensive demands on laboratory staff as well as the risk of human error, we have implemented an automated pipeline using a liquid-handling robot, Opentrons OT-2. This open source robotic platform lends itself to scalability and rapid deployment. The OT2 is modularly constructed and utilises widely available non-proprietary electronic components that are neither difficult nor expensive to repair or replace. Flexibility is offered through the Python Application Programming Interface (API), which simplifies custom protocol development. The additional laboratory automation described here, refers to the automatic determination of the individual positive result(s) and the de-bundling (or ungrouping) of pooled results into individual results using an automated web interface. The advantages of automated sample pooling include increased human-resource time (which can be better allocated for data analysis, experimental setup, etc.), the elimination of the possibility of human error (due to fatigue or distraction, for example), the reduction of costs associated with manual labour, and the flexibility to easily replicate or modify protocols based on the starting number of samples and desired pooling strategy.

## Results

### 3-d 27-sample hypercube experiments

**Figure 2:**
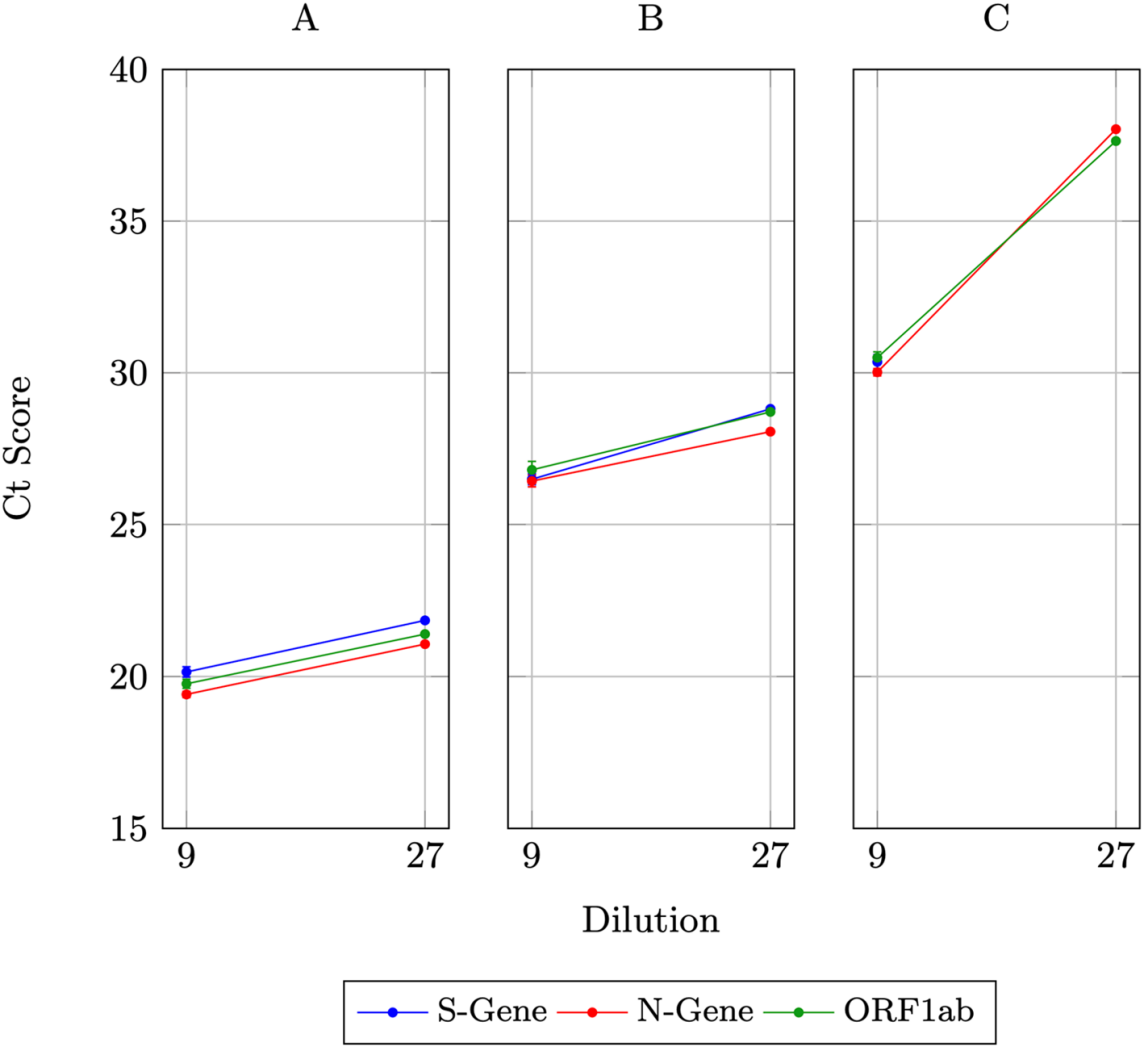
Detection of a single positive sample after ninefold and 27-fold dilution, in 3-d hypercube pooling. Error bars are shown for the 3 positive pools, where the positive sample is diluted ninefold.

The results of three 3-d hypercube experiments (Experiments A–C), in which a single positive sample is diluted up to 27-fold, are shown in Fig. 2. The plots are based on the Ct scores obtained for each of the three target genes (Supplementary Information Table S3).

The results show that the positive samples for each experiment can be successfully amplified and detected in the pool containing 27 samples and in each of the positive slices containing 9 samples each.

### 4-d 81-sample hypercube experiments

**Figure 3:**
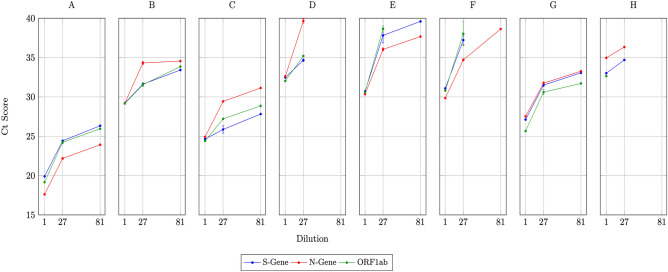
Detection of a single positive sample out of 81 for each of eight 4-d hypercube experiments (**A**–**H**). Ct scores are shown for: (a) the undiluted sample, (b) the mean and standard errors for the four positive slice pools which is diluted 27-fold, and (c) the hypercube pool in which the single positive is diluted 81-fold.

The results of eight 4-d hypercube experiments (Experiments A–H), in which a single positive sample is diluted up to 81-fold, are shown in Fig. 3. The plots are based on the Ct scores obtained for each of the three target genes (Supplementary Information Table S4).

The operational sensitivity of the pooled test procedure is decreased for samples with an initial (undiluted) Ct value exceeding 32 as indicated by experiments D and H, where amplification in the 81-pool samples was not successful (none of the target genes were detected).

### Efficiency—number of tests saved using the pooling method

We received approximately 121 samples per week over an 8 week period, for testing (i.e. 968 individual samples in total). Each week, three groups of either 27 or 81 pooled tests were performed (i.e. 24 sets of pooled tests in total). The starting point for each set of pooled tests is a single, group-level test, in which either 27 or 81 samples are pooled and then tested. Slice-level tests are conducted only if the group-level test is positive. Since all 24 group-level tests were negative, a greater than 40 (i.e. 986 divided by 24)-fold reduction in the number of tests used was achieved compared to individual testing.

### Laboratory automation

We created an automated and easy-to-use web interface to assist in the de-bundling of pooled testing results and the identification of the positive sample(s) found in a pool. The de-bundling module requires as input the analysis file generated from the pooled testing results and a list of sample identifiers. It produces a de-bundling report, which can be easily integrated into a laboratory information management system (LIMS) such as LabWare 7 (used in our laboratory), for individual result reporting.

To infer the positive sample, the user specifies the pool size, i.e. 27 or 81, the number of positive slices and their respective slice numbers. The application then uses this information to determine and output individual positive test results. Both applications, freely available at http://krisp2.ukzn.ac.za:8080/rega-pooling/pooling (Supplementary Information Figure. S3), were used for all the analysis and reporting of results described in this paper.

## Discussion

Diagnostic detection of SARS-CoV-2 is crucial for managing the pandemic^12,13^. The early identification and isolation of SARS-CoV-2 infected individuals remain a key strategy to interrupt community transmission. In this pilot study, we have successfully implemented hypercube pooling for frequent testing of a professional sports team. We show that this procedure reliably detects a single positive sample in a group of 81, provided the starting sample has a Ct ≤ 32. Whilst there is a need for further evaluation, particularly with samples with a wider range of Ct values and pools with multiple positive samples for different viral prevalence scenarios, these results indicate that the pooling strategy is indeed a promising approach for cost-efficient RT-PCR testing.

Other studies have demonstrated the successful implementation of pooled testing using groups of up to 5 and 8 samples each^8^. To our knowledge, this is the first study that has implemented the SARS-CoV-2 hypercube-based pooled testing strategy, using group sizes as large as 81.

We selected for analysis samples with Ct values that are typical for the population under study. For comparison, we also selected samples with Ct values that are one standard deviation away from the mean Ct value. We used historical data from the population of samples tested routinely in KZN to determine the distribution of Ct values present at various percentile ranges (Supplementary Information Figure S1). The asymmetrical (left-skewed) distribution of the data may be attributed to the population of samples that was used to generate the curve i.e. symptomatic people with active disease (and therefore lower Ct scores) presenting at health facilities.

The retention of the sensitivity of the pooled test procedure as the hypercube dimension size (and consequently the dilution) increases is an obvious concern.

Our results demonstrated no loss of assay sensitivity for samples with an initial (undiluted) Ct value ≤ 32 (Fig. 3). Samples with higher Ct values would typically have a lower viral load, which may be deemed as clinically and / or epidemiologically insignificant, since the infection is not likely to be contagious (a cut-off Ct > 30 can be associated with non-infectious samples)^14^.

Propagating SARS-CoV-2 from clinical samples can also be used as a valuable proxy for infectiousness. There are however, conflicting reports on the cut-off Ct value at which the virus is not cultivable, which can range anywhere from 30 to > 35^15,16^. In any event, high Ct values are expected to be associated with low infectivity^17^; the maximum viral load occurs during the onset of symptoms, when the risk of transmission is highest^18^. In respiratory samples, the viral load is highest during the initial stage of infection (patients in the early stages of infection usually have Ct values of 20–30 or less^16^), and reaches a peak in the second week, and then falls^19^. The pooling strategy we have implemented is therefore most beneficial for detecting asymptomatic or presymptomatic people who are on the viral load incline. In addition, the effects of the loss of assay sensitivity can be mitigated by implementing more frequent testing—a solution which could be more easily afforded given the demonstrated cost savings of the pooled testing strategy.

There have also been reports of inherent inaccuracy due to higher false negative rates associated with the pooled testing method^20^. Our method however, includes many consistency checks. For example, the detection of a single positive pool (corresponding to a hypercube slice) when all other pools are negative is indicative of a testing error^11^. This is especially beneficial when compared to approaches such as binary pooled testing^21^ which relies on repeated, sequential testing of the positive sample, and a single negative test can prematurely terminate the sequence of tests leading to a false negative result. Another cause of false negative results is sequence variation at primer or probe binding sites on the viral RNA^22^. In South Africa, a new SARS-CoV-2 lineage (501Y.V2) characterised by various mutations in the spike (S) gene has been reported^3^. However, there was no apparent loss of sensitivity for the S-gene in the RT-PCR assay used in this study.

We have developed and successfully implemented a pilot SARS-CoV-2 pooled testing strategy for a prominent South African rugby team. The successful implementation of pooled testing requires that 3 important criteria are met viz. (i) efficiency, (ii) sensitivity and (iii) operational feasibility^8^. Our study was successful in satisfying each of these criteria. Firstly, we achieved a > 40-fold gain in testing efficiency, and a corresponding reduction in cost, compared to individual testing. The cost reduction is most impressive when all individuals are negative, because 27 or 81 different samples can be determined to be negative by using just one qPCR reaction, in a 3-d or 4-d hypercube, respectively. This was the case with the screening of a South African rugby team at the KRISP laboratory, where, during the first 8 weeks, all of the samples were tested using hypercubes of sizes 27 or 81, and found to be negative. Only from the 9th week was an additional round of slice testing required. Secondly, no significant loss of sensitivity for samples with appreciable viral loads (Ct ≤ 32) was observed. Thirdly, we validated our pooling workflow, i.e., by using liquid-handling robots to automated sample pooling and software applications to automate the identification of positive samples.

There are more conventional methods for high-throughput diagnostic testing which are available. For example, SARS-CoV-2 lateral flow antigen (LFA) tests are point-of-care rapid tests that can be used for large-scale screening. Although LFAs may be appealing because they are cheap, since they do not require a laboratory with specialized equipment or personnel and can provide results in 15–30 min, they do present some limitations; the sensitivity of this type of technology can vary considerably around an average sensitivity of 56.2% (95% CI 29.5 to 79.8%)^23^, thereby decreasing its utility in screening certain populations such as health-care workers and other front-line personnel. We have performed a detailed cost comparison of the hypercube-based pooled testing strategy compared to the LFAs. We estimate that at prevalences below 0.43%, it costs over 6 times more to achieve the reliable detection (i.e. a detection probability of at least 99.9%) of SARS-CoV-2 infectious individuals by using LFAs compared to hypercube testing (Supplementary Information—Cost comparison of hypercube-based pooled tests vs. lateral flow antigen tests). Despite their higher cost for reliable detection, LFAs may be used to complement hypercube-based testing, particularly when quick results are needed and the lower sensitivity of the assay is not considered problematic.

The pooled testing strategy and downstream automation protocol described in this study have together led to a significant reduction in cost, kit usage and turnaround time in our laboratory. While we demonstrated its successful application for frequent testing of a professional sports team, this approach can potentially be applied for screening other low prevalence, asymptomatic population groups. Further evaluation of this approach in different populations and epidemic settings is desirable.

## Methods

### SARS-CoV-2 clinical diagnostics

The standard protocol used for SARS-CoV-2 detection at the KRISP Laboratory, University of KwaZulu-Natal, is to extract viral ribonucleic acid (RNA) from nasopharyngeal and / or oropharyngeal swab samples. This is followed by RT-PCR (TaqPath COVID-19 CE-IVD RT-PCR kit, ThermoFisher Scientific, MA, USA) for the detection of three SARS-CoV-2 target genes (viz. S-gene, N-gene and ORF1ab). A sample is considered “positive” if at least 2 out of the 3 target genes are amplified with an above-background fluorescence signal, cycle threshold, Ct ≤ 40 PCR cycles. A sample is considered “inconclusive” if 1 out of the 3 target genes is positive (Ct ≤ 40) and should then be repeat tested. A sample is considered “negative” if none of the 3 target genes is amplified. Swab samples collected in viral transport medium (VTM) for routine SARS-CoV-2 surveillance in KwaZulu-Natal, South Africa were used in this study. All experiments were conducted in accordance with relevant local guidelines and regulations.

Historical data from a population of approximately 1200 samples tested routinely in KZN was used to determine the distribution of Ct values present at various percentile ranges (Supplementary Information Figure S1). Positive samples used for each of the experiments were chosen based on their respective Ct scores to represent different quantiles of the Ct value distribution.

Pooling experiments were conducted to verify the successful detection of a positive sample embedded inside: (1) a 3-d hypercube containing 26 other, negative samples; and (2) a 4-d hypercube containing 80 other, negative samples.

### 27-sample, 3-d hypercube experiments

Three positive samples were used in three independent pooling tests to evaluate proof-of-concept and determine whether these samples would still result in successful PCR amplification when diluted 27- (in group-level pools) and 9- (in slice-level pools) fold using 3-d hypercubes.

First, for each positive sample, a group-level pool containing 27 samples was created. Each positive sample was combined with 26 negative swab samples as follows: sample tubes were labelled from 1 to 27. Samples were added to each of the labelled tubes, with the positive sample inserted blindly, i.e. in a position of the hypercube not known to the person performing the pooling experiments or the analysis of results. Each sample was vortexed briefly and 200 µl was manually pipetted into a separate collection tube containing the pooled sample. The collection tube (with a total volume of 5400 µl) was then briefly vortexed and 200 µl was used for paramagnetic bead-based RNA extraction using the Chemagic360 automated extraction system (PerkinElmer Inc., Cat.# CMG-1049, MA, USA). The purified nucleic acid was eluted in 100 µl of elution buffer. 10 µl of RNA was then used in the RT-PCR reaction (TaqPath™ COVID-19 CE-IVD RT-PCR Kit, Thermo Fisher Scientific).

Group-level pools that were positive were sub-pooled into 9 slice-level pools, containing 9 samples each (Supplementary Information—Table S1). Each sample found in the positive, group-level pool was represented in 3 different slice-level pools.

### 81-sample, 4-d hypercube experiments

As in the 3-d experiments, positive samples were chosen to represent the different quantiles of the Ct value distribution that is typical of our study population (Supplementary Information Fig. S1).

One positive swab sample was combined with 80 negative swab samples in each of eight experiments (Experiments A–H). For each experiment, sample tubes were labelled from 1 to 81, and each of the respective positive samples was inserted blindly into a position within the 81-sample hypercube. Each sample was vortexed briefly and 200 µl was added to a separate collection tube containing the pooled sample. The collection tube (with a total volume of 16 200 µl for each experiment) was then briefly vortexed and 200 µl was used for RNA extraction and subsequent RT-PCR, as described above. For each experiment, the 81-sample pool was further sub-pooled into 12 slices containing 27 samples each (Supplementary Information—Table S2). Each individual sample was therefore represented in 4 different slices in each experiment.

Based on the consistent amplification of the positive sample across the respective slices in each of the 3-d and 4-d hypercube experiments, the location of the positive sample could be deduced.

### Laboratory robotics

The robotics work flow is summarised in the following steps (Supplementary Information Fig. S2):

1. A new 200 µl tip is collected by the single-channel 300 µl electronic pipette.
2. The electronic pipette draws 200 µl from the sample rack for the current slice iteration.
3. The electronic pipette dispenses the sample in the relevant sample sub-pool.
4. The tip is discarded into the robot’s trash.

The programming codes for the 3-d and 4-d pooling experiments using the OT-2 liquid handling robot is available at https://github.com/krisp-kwazulu-natal/efficient-SARS-CoV-2-pooled-testing-strategy-code.

### Software for the de-bundling and inference of positive results

Custom scripts were implemented in Python for the de-bundling and inference of positive results from a pool of 27 or 81 samples. When inferring positive results, our scripts take into account degenerates and can infer more than one positive sample per pool at low prevalence. For ease of use, we developed a user-friendly, web-based front end using the Java Web Toolkit (Jwt), an open-source, robust Java web front end (GUI; http://webtoolkit.eu/jwt) library. Minimal user interaction is required as user interaction is limited to uploading the files to be processed or providing pool details in the case of inference. A report detailing the de-bundling results is provided to the user once processing of the data is complete. Our de-bundling and inference software is freely available and can be accessed at the URL: http://krisp2.ukzn.ac.za:8080/rega-pooling/pooling. The programming scripts for the software we developed are available at https://github.com/krisp-kwazulu-natal/efficient-SARS-CoV-2-pooled-testing-strategy-code.

### Ethics declarations

We used de-identified remnant nasopharyngeal and oropharyngeal swab samples from patients tested positive for SARS-CoV-2 by RT–qPCR from public health laboratories in South Africa. The project was approved by University of KwaZulu-Natal Biomedical Research Ethics Committee (Protocol reference no. BREC/00,001,195/2020; project title: COVID-19 transmission and natural history in KwaZulu-Natal, South Africa: epidemiological investigation to guide prevention and clinical care).

## Supplementary Information

Supplementary Information.
