# Supplementary material for: Implementation of an efficient SARS-CoV-2 specimen pooling strategy for high throughput diagnostic testing
Source: Sci Rep. 2021 Sep 7;11:17793. doi: 10.1038/s41598-021-96934-z (PMC8423848; doi:10.1038/s41598-021-96934-z)
Supplement: Supplementary file 1 — Supplementary Information. [file 41598_2021_96934_MOESM1_ESM.docx]

**Supplementary information**

Table S1. Slicing pattern for sub-pools using a 27-sample (3 dimensional) hypercube experiment

| Slice 1 sub-pool | Slice 2 sub-pool | Slice 3 sub-pool | Slice 4 sub-pool | Slice 5 sub-pool | Slice 6 sub-pool | Slice 7 sub-pool | Slice 8 sub-pool | Slice 9 sub-pool |
| --- | --- | --- | --- | --- | --- | --- | --- | --- |
| 1 | 10 | 19 | 1 | 4 | 7 | 1 | 2 | 3 |
| 2 | 11 | 20 | 2 | 5 | 8 | 4 | 5 | 6 |
| 3 | 12 | 21 | 3 | 6 | 9 | 7 | 8 | 9 |
| 4 | 13 | 22 | 10 | 13 | 16 | 10 | 11 | 12 |
| 5 | 14 | 23 | 11 | 14 | 17 | 13 | 14 | 15 |
| 6 | 15 | 24 | 12 | 15 | 18 | 16 | 17 | 18 |
| 7 | 16 | 25 | 19 | 22 | 25 | 19 | 20 | 21 |
| 8 | 17 | 26 | 20 | 23 | 26 | 22 | 23 | 24 |
| 9 | 18 | 27 | 21 | 24 | 27 | 25 | 26 | 27 |

Table S2. Slicing pattern for sub-pools using an 81-sample (4 dimensional) hypercube experiment

| Slice 1 sub-pool | Slice 2 sub-pool | Slice 3 sub-pool | Slice 4 sub-pool | Slice 5 sub-pool | Slice 6 sub-pool | Slice 7 sub-pool | Slice 8 sub-pool | Slice 9 sub-pool | Slice 10 sub-pool | Slice 11 sub-pool | Slice 12 sub-pool |
| --- | --- | --- | --- | --- | --- | --- | --- | --- | --- | --- | --- |
| 1 | 28 | 55 | 1 | 10 | 19 | 1 | 4 | 7 | 1 | 2 | 3 |
| 2 | 29 | 56 | 2 | 11 | 20 | 2 | 5 | 8 | 4 | 5 | 6 |
| 3 | 30 | 57 | 3 | 12 | 21 | 3 | 6 | 9 | 7 | 8 | 9 |
| 4 | 31 | 58 | 4 | 13 | 22 | 10 | 13 | 16 | 10 | 11 | 12 |
| 5 | 32 | 59 | 5 | 14 | 23 | 11 | 14 | 17 | 13 | 14 | 15 |
| 6 | 33 | 60 | 6 | 15 | 24 | 12 | 15 | 18 | 16 | 17 | 18 |
| 7 | 34 | 61 | 7 | 16 | 25 | 19 | 22 | 25 | 19 | 20 | 21 |
| 8 | 35 | 62 | 8 | 17 | 26 | 20 | 23 | 26 | 22 | 23 | 24 |
| 9 | 36 | 63 | 9 | 18 | 27 | 21 | 24 | 27 | 25 | 26 | 27 |
| 10 | 37 | 64 | 28 | 37 | 46 | 28 | 31 | 34 | 28 | 29 | 30 |
| 11 | 38 | 65 | 29 | 38 | 47 | 29 | 32 | 35 | 31 | 32 | 33 |
| 12 | 39 | 66 | 30 | 39 | 48 | 30 | 33 | 36 | 34 | 35 | 36 |
| 13 | 40 | 67 | 31 | 40 | 49 | 37 | 40 | 43 | 37 | 38 | 39 |
| 14 | 41 | 68 | 32 | 41 | 50 | 38 | 41 | 44 | 40 | 41 | 42 |
| 15 | 42 | 69 | 33 | 42 | 51 | 39 | 42 | 45 | 43 | 44 | 45 |
| 16 | 43 | 70 | 34 | 43 | 52 | 46 | 49 | 52 | 46 | 47 | 48 |
| 17 | 44 | 71 | 35 | 44 | 53 | 47 | 50 | 53 | 49 | 50 | 51 |
| 18 | 45 | 72 | 36 | 45 | 54 | 48 | 51 | 54 | 52 | 53 | 54 |
| 19 | 46 | 73 | 55 | 64 | 73 | 55 | 58 | 61 | 55 | 56 | 57 |
| 20 | 47 | 74 | 56 | 65 | 74 | 56 | 59 | 62 | 58 | 59 | 60 |
| 21 | 48 | 75 | 57 | 66 | 75 | 57 | 60 | 63 | 61 | 62 | 63 |
| 22 | 49 | 76 | 58 | 67 | 76 | 64 | 67 | 70 | 64 | 65 | 66 |
| 23 | 50 | 77 | 59 | 68 | 77 | 65 | 68 | 71 | 67 | 68 | 69 |
| 24 | 51 | 78 | 60 | 69 | 78 | 66 | 69 | 72 | 70 | 71 | 72 |
| 25 | 52 | 79 | 61 | 70 | 79 | 73 | 76 | 79 | 73 | 74 | 75 |
| 26 | 53 | 80 | 62 | 71 | 80 | 74 | 77 | 80 | 76 | 77 | 78 |
| 27 | 54 | 81 | 63 | 72 | 81 | 75 | 78 | 81 | 79 | 80 | 81 |


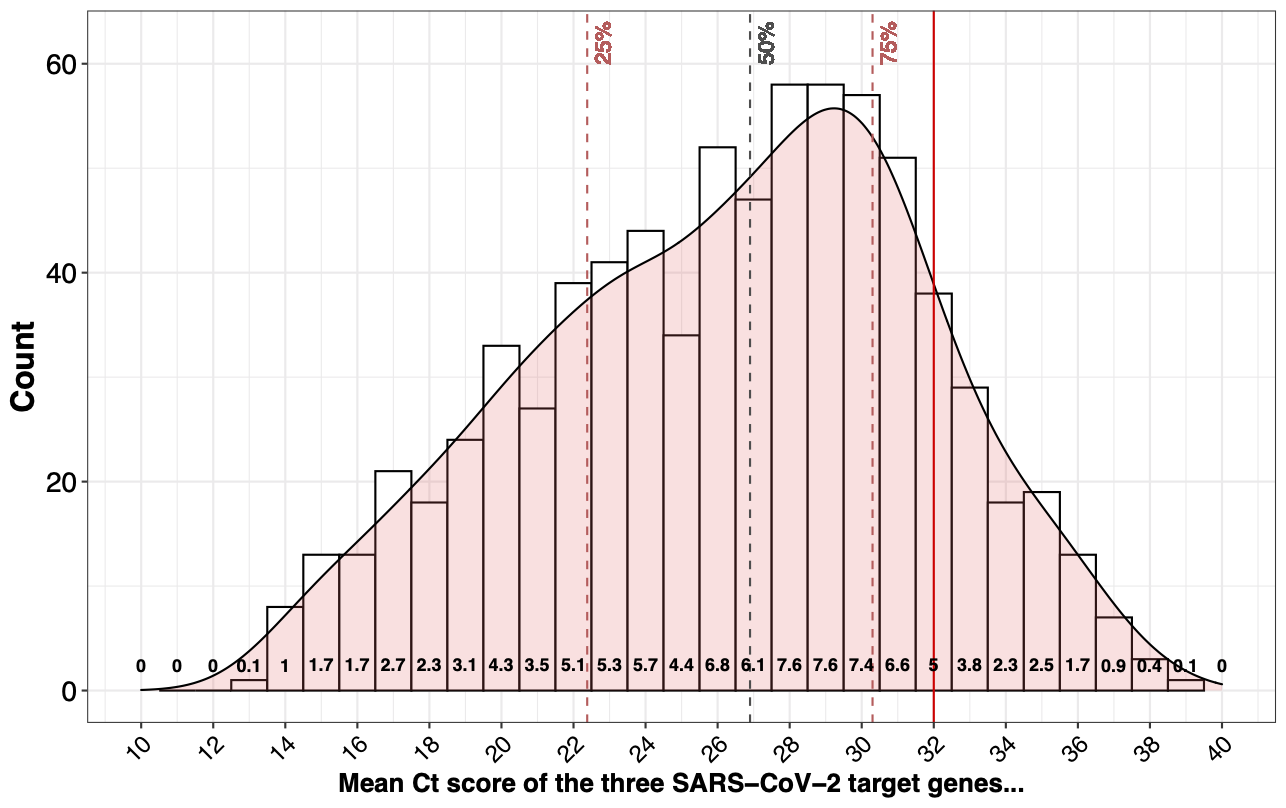


Fig. S1. Distribution of Cycle Threshold (Ct) scores from population of samples tested in KwaZulu-Natal; the figures inside the bars represent the percentage of samples that fall within this Ct score range. The solid red vertical line represents the Ct at which sensitivity of the assay is compromised (Ct ≥ 32).

Table S3. 27-sample 3-d hypercube results

| **Experiment ID** | **Description** | **S-gene Ct** | **N-gene Ct** | **ORF1ab Ct** | **Result** |
| --- | --- | --- | --- | --- | --- |
| A | 27-pool sample | 21.85 | 21.07 | 21.4 | Positive |
|  | 9-pool slice 1 | Undetermined | Undetermined | Undetermined | Negative |
|  | 9-pool slice 2 | Undetermined | Undetermined | Undetermined | Negative |
|  | 9-pool slice 3 | 19.99 | 19.22 | 19.55 | Positive |
|  | 9-pool slice 4 | Undetermined | Undetermined | Undetermined | Negative |
|  | 9-pool slice 5 | 19.92 | 19.39 | 19.63 | Positive |
|  | 9-pool slice 6 | Undetermined | Undetermined | Undetermined | Negative |
|  | 9-pool slice 7 | Undetermined | Undetermined | Undetermined | Negative |
|  | 9-pool slice 8 | Undetermined | Undetermined | Undetermined | Negative |
|  | 9-pool slice 9 | 20.53 | 19.62 | 20.09 | Positive |
|  | Standard deviation | 0.33 | 0.20 | 0.29 | |
| B | 27-pool sample | 28.81 | 28.06 | 28.71 | Positive |
|  | 9-pool slice 1 | Undetermined | Undetermined | Undetermined | Negative |
|  | 9-pool slice 2 | Undetermined | Undetermined | Undetermined | Negative |
|  | 9-pool slice 3 | 26.94 | 26.92 | 27.51 | Positive |
|  | 9-pool slice 4 | Undetermined | Undetermined | Undetermined | Negative |
|  | 9-pool slice 5 | 26.4 | 26.14 | 26.39 | Positive |
|  | 9-pool slice 6 | Undetermined | Undetermined | Undetermined | Negative |
|  | 9-pool slice 7 | Undetermined | Undetermined | Undetermined | Negative |
|  | 9-pool slice 8 | Undetermined | Undetermined | Undetermined | Negative |
|  | 9-pool slice 9 | 26.14 | 26.22 | 26.51 | Positive |
|  | Standard deviation | 0.41 | 0.43 | 0.61 | |
| C | 27-pool sample | Undetermined | 38.03 | 37.64 | Positive |
|  | 9-pool slice 1 | Undetermined | Undetermined | Undetermined | Negative |
|  | 9-pool slice 2 | Undetermined | Undetermined | Undetermined | Negative |
|  | 9-pool slice 3 | 30.91 | 30.31 | 30.9 | Positive |
|  | 9-pool slice 4 | Undetermined | Undetermined | Undetermined | Negative |
|  | 9-pool slice 5 | 29.97 | 29.77 | 30.54 | Positive |
|  | 9-pool slice 6 | Undetermined | Undetermined | Undetermined | Negative |
|  | 9-pool slice 7 | Undetermined | Undetermined | Undetermined | Negative |
|  | 9-pool slice 8 | Undetermined | Undetermined | Undetermined | Negative |
|  | 9-pool slice 9 | 30.21 | 29.97 | 30.06 | Positive |
|  | Standard deviation | 0.49 | 0.27 | 0.42 | |

Pool = 27-fold dilution; Slice = 9-fold dilution

Based on the consistent amplification of the positive sample across the respective slices, the location of the positive sample can be deduced to be in position 24 within each of the three 3-d hypercubes.

Table S4. 81- sample 4-d hypercube results

| **Experiment ID** | **Description** | **S-gene Ct** | **N-gene Ct** | **ORF1ab Ct** | **Result** |
| --- | --- | --- | --- | --- | --- |
| A | Undiluted positive | 19.9 | 17.62 | 19.16 | Positive |
|  | 81-pool sample | 26.33 | 23.92 | 25.95 | Positive |
|  | 27-pool slice 1 | Undetermined | Undetermined | Undetermined | Negative |
|  | 27-pool slice 2 | Undetermined | Undetermined | Undetermined | Negative |
|  | 27-pool slice 3 | 24.24 | 22.04 | 23.92 | Positive |
|  | 27-pool slice 4 | 24.52 | 22.35 | 24.55 | Positive |
|  | 27-pool slice 5 | Undetermined | Undetermined | Undetermined | Negative |
|  | 27-pool slice 6 | Undetermined | Undetermined | Undetermined | Negative |
|  | 27-pool slice 7 | Undetermined | Undetermined | Undetermined | Negative |
|  | 27-pool slice 8 | 24.67 | 22.37 | 24.52 | Positive |
|  | 27-pool slice 9 | Undetermined | Undetermined | Undetermined | Negative |
|  | 27-pool slice 10 | 24.25 | 21.94 | 23.92 | Positive |
|  | 27-pool slice 11 | Undetermined | Undetermined | Undetermined | Negative |
|  | 27-pool slice 12 | Undetermined | Undetermined | Undetermined | Negative |
|  | Standard deviation | 0.21 | 0.22 | 0.36 | |
| B | Undiluted positive | 29.21 | 29.20 | 29.16 | Positive |
|  | 81-pool sample | 33.42 | 34.55 | 33.85 | Positive |
|  | 27-pool slice 1 | 31.23 | 34.29 | 31.35 | Positive |
|  | 27-pool slice 2 | Undetermined | Undetermined | Undetermined | Negative |
|  | 27-pool slice 3 | Undetermined | Undetermined | Undetermined | Negative |
|  | 27-pool slice 4 | Undetermined | Undetermined | Undetermined | Negative |
|  | 27-pool slice 5 | 31.68 | 34.92 | 31.53 | Positive |
|  | 27-pool slice 6 | Undetermined | Undetermined | Undetermined | Negative |
|  | 27-pool slice 7 | Undetermined | Undetermined | Undetermined | Negative |
|  | 27-pool slice 8 | Undetermined | Undetermined | Undetermined | Negative |
|  | 27-pool slice 9 | 32.05 | 34.04 | 32.23 | Positive |
|  | 27-pool slice 10 | Undetermined | Undetermined | Undetermined | Negative |
|  | 27-pool slice 11 | 31.47 | 34 | 31.08 | Positive |
|  | 27-pool slice 12 | Undetermined | Undetermined | Undetermined | Negative |
|  | Standard deviation | 0.35 | 0.42 | 0.49 | |
| C | Undiluted positive | 24.64 | 24.91 | 24.41 | Positive |
|  | 81-pool sample | 27.82 | 31.13 | 28.86 | Positive |
|  | 27-pool slice 1 | Undetermined | Undetermined | Undetermined | Negative |
|  | 27-pool slice 2 | 25.83 | 29.48 | 27.22 | Positive |
|  | 27-pool slice 3 | Undetermined | Undetermined | Undetermined | Negative |
|  | 27-pool slice 4 | Undetermined | Undetermined | Undetermined | Negative |
|  | 27-pool slice 5 | Undetermined | Undetermined | Undetermined | Negative |
|  | 27-pool slice 6 | 26.86 | 29.47 | 27.17 | Positive |
|  | 27-pool slice 7 | 26.11 | 29.44 | 27.21 | Positive |
|  | 27-pool slice 8 | Undetermined | Undetermined | Undetermined | Negative |
|  | 27-pool slice 9 | Undetermined | Undetermined | Undetermined | Negative |
|  | 27-pool slice 10 | 24.64 | 29.34 | 27.25 | Positive |
|  | 27-pool slice 11 | Undetermined | Undetermined | Undetermined | Negative |
|  | 27-pool slice 12 | Undetermined | Undetermined | Undetermined | Negative |
|  | Standard deviation | 0.92 | 0.06 | 0.03 | |
| D | Undiluted positive | 32.46 | 32.60 | 32.03 | Positive |
|  | 81-pool sample | Undetermined | Undetermined | Undetermined | Negative |
|  | 27-pool slice 1 | Undetermined | Undetermined | Undetermined | Negative |
|  | 27-pool slice 2 | Undetermined | Undetermined | Undetermined | Negative |
|  | 27-pool slice 3 | Undetermined | Undetermined | 35.2 | Inconclusive |
|  | 27-pool slice 4 | 34.82 | 39.37 | Undetermined | Positive |
|  | 27-pool slice 5 | Undetermined | Undetermined | Undetermined | Negative |
|  | 27-pool slice 6 | Undetermined | Undetermined | Undetermined | Negative |
|  | 27-pool slice 7 | Undetermined | Undetermined | Undetermined | Negative |
|  | 27-pool slice 8 | 34.5 | 39.93 | Undetermined | Positive |
|  | 27-pool slice 9 | Undetermined | Undetermined | Undetermined | Negative |
|  | 27-pool slice 10 | Undetermined | Undetermined | Undetermined | Negative |
|  | 27-pool slice 11 | Undetermined | Undetermined | Undetermined | Negative |
|  | 27-pool slice 12 | Undetermined | Undetermined | Undetermined | Negative |
|  | Standard deviation | 0.23 | 0.40 | NA | |
| E | Undiluted positive | 30.7 | 30.35 | 30.6 | Positive |
|  | 81-pool sample | 39.59 | 37.67 | Undetermined | Positive |
|  | 27-pool slice 1 | 39.9 | 36.56 | 38.46 | Positive |
|  | 27-pool slice 2 | Undetermined | Undetermined | Undetermined | Negative |
|  | 27-pool slice 3 | Undetermined | Undetermined | Undetermined | Negative |
|  | 27-pool slice 4 | 38.97 | 35.88 | 39.44 | Positive |
|  | 27-pool slice 5 | Undetermined | Undetermined | Undetermined | Negative |
|  | 27-pool slice 6 | Undetermined | Undetermined | Undetermined | Negative |
|  | 27-pool slice 7 | Undetermined | Undetermined | Undetermined | Negative |
|  | 27-pool slice 8 | Undetermined | Undetermined | Undetermined | Negative |
|  | 27-pool slice 9 | 36.52 | 35.7 | 37.61 | Positive |
|  | 27-pool slice 10 | Undetermined | Undetermined | Undetermined | Negative |
|  | 27-pool slice 11 | 35.88 | 36 | 39.03 | Positive |
|  | 27-pool slice 12 | Undetermined | Undetermined | Undetermined | Negative |
|  | Standard deviation | 1.92 | 0.37 | 0.79 | |
| F | Undiluted positive | 31.08 | 29.85 | 30.8 | Positive |
|  | 81-pool sample | Undetermined | 38.63 | Undetermined | Inconclusive |
|  | 27-pool slice 1 | Undetermined | Undetermined | Undetermined | Negative |
|  | 27-pool slice 2 | Undetermined | Undetermined | Undetermined | Negative |
|  | 27-pool slice 3 | 37.33 | 34.55 | 35.78 | Positive |
|  | 27-pool slice 4 | Undetermined | Undetermined | Undetermined | Negative |
|  | 27-pool slice 5 | 38.79 | 34.38 | 37.11 | Positive |
|  | 27-pool slice 6 | Undetermined | Undetermined | Undetermined | Negative |
|  | 27-pool slice 7 | 36.86 | 35 | 36.75 | Positive |
|  | 27-pool slice 8 | Undetermined | Undetermined | Undetermined | Negative |
|  | 27-pool slice 9 | Undetermined | Undetermined | Undetermined | Negative |
|  | 27-pool slice 10 | 35.88 | 34.88 | 42.48 | Positive |
|  | 27-pool slice 11 | Undetermined | Undetermined | Undetermined | Negative |
|  | 27-pool slice 12 | Undetermined | Undetermined | Undetermined | Negative |
|  | Standard deviation | 1.21 | 0.29 | 3.02 | |
| G | Undiluted positive | 27.12 | 27.52 | 25.66 | Positive |
|  | 81-pool sample | 33.06 | 33.26 | 31.71 | Positive |
|  | 27-pool slice 1 | Undetermined | Undetermined | Undetermined | Negative |
|  | 27-pool slice 2 | Undetermined | Undetermined | Undetermined | Negative |
|  | 27-pool slice 3 | 30.96 | 31.44 | 30.31 | Positive |
|  | 27-pool slice 4 | 31.47 | 31.47 | 29.91 | Positive |
|  | 27-pool slice 5 | Undetermined | Undetermined | Undetermined | Negative |
|  | 27-pool slice 6 | Undetermined | Undetermined | Undetermined | Negative |
|  | 27-pool slice 7 | Undetermined | Undetermined | Undetermined | Negative |
|  | 27-pool slice 8 | 31.65 | 31.86 | 30.72 | Positive |
|  | 27-pool slice 9 | Undetermined | Undetermined | Undetermined | Negative |
|  | 27-pool slice 10 | Undetermined | Undetermined | Undetermined | Negative |
|  | 27-pool slice 11 | 31.91 | 32.18 | 31.44 | Positive |
|  | 27-pool slice 12 | Undetermined | Undetermined | Undetermined | Negative |
|  | Standard deviation | 0.40 | 0.35 | 0.65 | |
| H | Undiluted positive | 33.01 | 34.96 | 32.65 | Positive |
|  | 81-pool sample | Undetermined | Undetermined | Undetermined | Negative |
|  | 27-pool slice 1 | Undetermined | Undetermined | Undetermined | Negative |
|  | 27-pool slice 2 | 34.70 | 36.34 | Undetermined | Positive |
|  | 27-pool slice 3 | Undetermined | Undetermined | Undetermined | Negative |
|  | 27-pool slice 4 | Undetermined | Undetermined | Undetermined | Negative |
|  | 27-pool slice 5 | Undetermined | Undetermined | Undetermined | Negative |
|  | 27-pool slice 6 | Undetermined | Undetermined | Undetermined | Negative |
|  | 27-pool slice 7 | Undetermined | Undetermined | Undetermined | Negative |
|  | 27-pool slice 8 | Undetermined | Undetermined | Undetermined | Negative |
|  | 27-pool slice 9 | Undetermined | Undetermined | Undetermined | Negative |
|  | 27-pool slice 10 | Undetermined | Undetermined | Undetermined | Negative |
|  | 27-pool slice 11 | Undetermined | Undetermined | Undetermined | Negative |
|  | 27-pool slice 12 | Undetermined | Undetermined | Undetermined | Negative |
|  | Standard deviation | NA | NA | NA | |

Pool = 81-fold dilution; Slice = 27-fold dilution


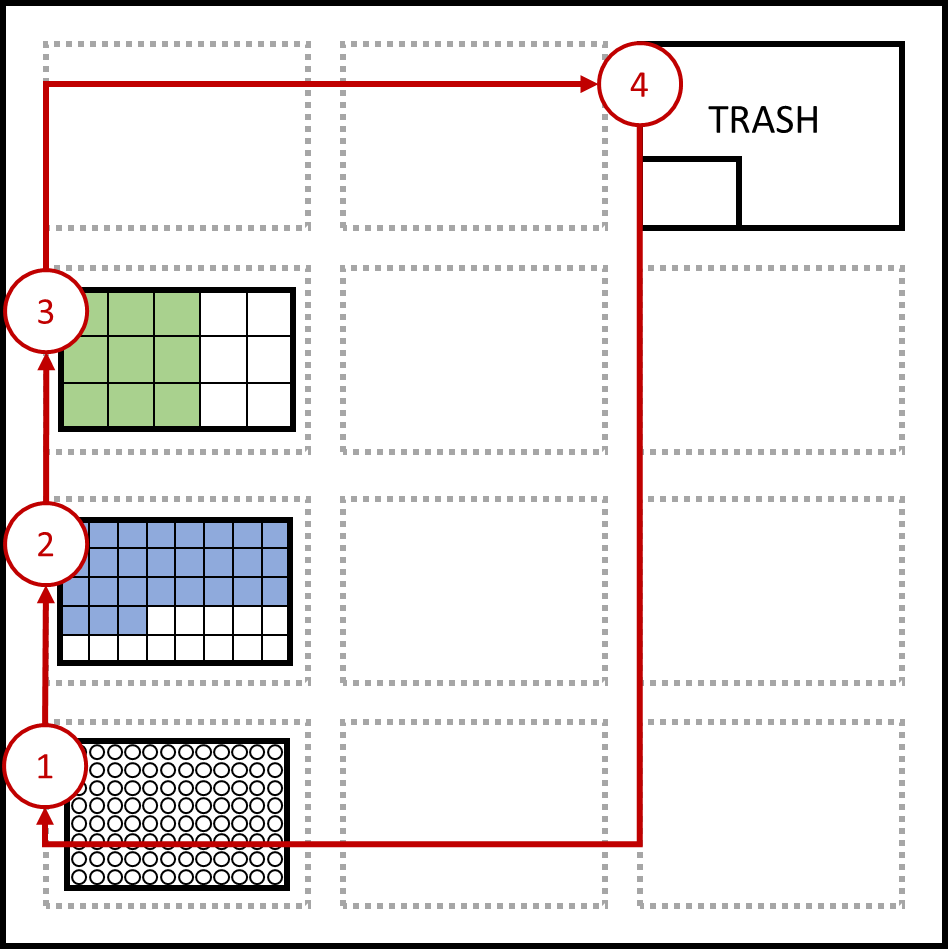


Fig. S2. Top view of the Opentrons2 liquid-handling robot and electronic pipette workflow path: position 1 – pipette tips; position 2 – source plate containing individual samples; position 3 – target plate containing sample pools; position 4 – trash compartment to discard used pipette tips


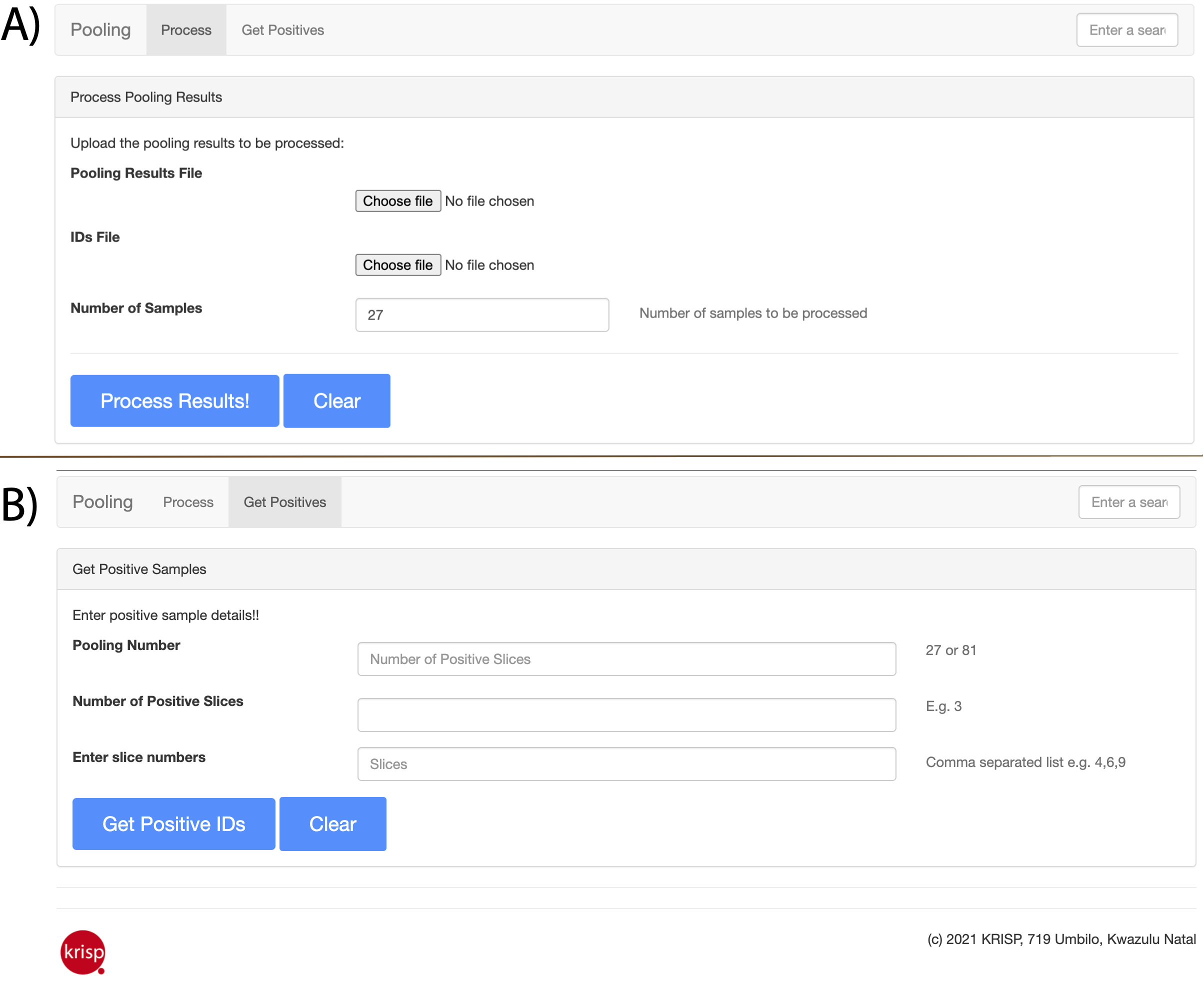


Fig. S3. Automated web application for processing pooling results. (A) Interface for inference of positive sample(s) in the pool. (B) Interface for de-bundling of pooled results into individual results.

**Cost comparison of hypercube-based pooled tests vs. lateral flow antigen tests**

Here we answer the following question: how much more will it cost to detect infectious individuals with a defined level of accuracy by using a lateral flow antigen (LFA) test vs. a hypercube-based (HC) pooled test with ≤81 samples per pool?

To illustrate the analytical framework that we will use, we denote by *C* = (0, 40] the range of possible Ct values that an infected individual can have. Suppose that an individual is infectious when their Ct value is within an interval $I\subset C$. We assume that individuals are infectious when their Ct value is below 30 *(11, 20)*, so we set *I* = (0, 30).

Now, let $\left\{ U_{i} \right\}$, $U_{i}=(c_{i-1},c_{i}]$, *i* = 1,2,…,*n*, be a set of sub-intervals induced by a finite partition $\left\{ c_{i} \right\}$ of the interval *C*, with 0 = *c*_1_ < *c*_2_ <$\cdot\cdot\cdot$ < *c*_n_ = 40. Let the probability that a particular testing method will produce a positive result when the Ct value is in the interval $U_{i}$ be denoted *p*_i_. Then, given an individual’s Ct trajectory the probability that the individual will test positive while infectious is given by

$p_{\text{pos}}=\sum_{i=1}^{n} \frac{\left| U_{i}^{t}\bigcap I^{t} \right|}{\left| I^{t} \right|}p_{i}$, (1)

where |*I*| denotes the length of the interval *I*, $\bigcap$denotes the intersection of intervals, and $I^{t}$ and $U_{i}^{t}$ are the time intervals for which the Ct values are in the corresponding range. Equation (1) follows from the assumption that an individual can be tested at any time point, chosen uniformly at random.

If the length of the infectious period is *t_I_* days, then infectiousness will be detected with probability *p*_pos_ if tests are conducted every *t_I_* days. If *p*_pos_ < 1, then it may be necessary to increase the frequency of testing to above 1/*t_I_*  day^-1^ in order to detect infectiousness with a sufficiently high probability.

Suppose that two testing methods A and B require testing frequencies of$f_{q}^{A}$ and $f_{q}^{B}$, respectively, to achieve a detection probability *q*. In addition, suppose that the testing cost per individual is *x*_A_ (respectively *x*_B_) for method A (respectively B). Then, the ratio of the cost required to achieve a detection probability *q* using method A vs. method B is given by

$r=\frac{f_{q}^{A}x_{A}}{f_{q}^{B}x_{B}}$. (2)

To compare the cost required to achieve a desired detection probability by using HC vs. LFA, we need the following information: (i) Sample Ct trajectories from infected individuals; (ii) the probability that a given method will produce a positive test as a function of the Ct value; and (iii) the testing cost per individual.

For (i), we use the posterior distribution of Ct trajectories generated by a mathematical model that was previously parametrized using experimental data *(21)*. A representative output from this model is shown in Fig. S4.


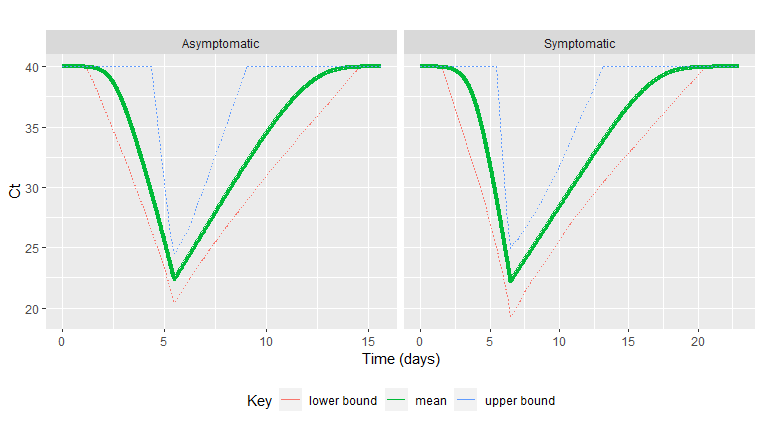


Fig. S4. Ct trajectories for asymptomatic (left panel) and symptomatic (right panel) individuals simulated using a mathematical model based on experimental data *(21)*. This model is conservative in allowing a sharp spike of infectiousness, so that the period of infectiousness is relatively brief. The mean trajectory calculated from 1000 simulations is shown together with envelopes (dotted lines) showing lower and upper 95% credible bounds for these trajectories at each time.

In addition, for (ii) and (iii), we make the following assumptions:

1) Based on data collected in our lab (see main text) and elsewhere *(21)*, for simplicity we assume that an HC test that dilutes positive samples by 81-fold produces a positive result 100% of the time when the positive sample’s Ct value is below 30. For LFA, we use the following sensitivity estimates *(20)*, which are optimistic in the light of published data *(22, 23)*: LFA yields a positive result 95% of the time when the Ct value is less than 27, and 65% of the time when the Ct value is between 27 and 30.

2) We assume that LFA costs USD 5 per individual *(24)*. The cost of each PCR test is about USD 56. The cost of HC is calculated as USD 56 times the expected number of tests per individual, which depends on the viral prevalence. At a viral prevalence that is just small enough to allow 81 samples to be pooled (i.e. a prevalence of 0.35/81 = 0.43%) the cost is USD 3.4. The cost per individual is lower at viral prevalences less than 0.43%.

Using the calculated posterior distribution of Ct trajectories (Fig. 5) *(21)*; we find that the mean length of the infectious period is 4.16$\pm$0.75 days for asymptomatic individuals and 5.8$\pm$1.03 days for symptomatic individuals. In addition, using Equation (1), we estimate that HC testing will detect 100% of infectious individuals if applied once during the infectious period, an average of once every 4 days for asymptomatics and once every 6 days for symptomatics. In contrast, LFA will detect only 83% of infectious individuals under the same conditions. We estimate that, in order to detect infection with a probability of 99.9%, an asymptomatic infectious individual will have to be tested with LFA up to 4 times during the infectious period, *i.e.,* daily. This is equivalent to the testing frequency calculated using the following simple formula based on the assumption that test outcomes are Bernoulli random variables:

$f_{q}^{\text{LFA}}=\frac{\text{log}\left( 1-q \right)}{t_{I} \text{log}\left( 1- p_{\text{pos}} \right)}$ day^-1^, (3)

where *q* = 0.999, *p*_pos_ = 0.83, and *t*_1_ = 4 days.

Therefore, using Equation (2), we estimate that, at a viral prevalence of 0.43%, it will cost 6 times more to detect infectious individuals with a 99.9% probability using LFA compared to using HC. The cost advantage of HC relative to LFA increases as the viral prevalence falls. Note that in this comparison we have neglected the possibility of detecting infection prior to the Ct score rising above 30. Since HC is more sensitive than LFA, it is more likely to succeed in detecting infection early, adding to its relative advantage.

**Supplementary Information References**

1. Singanayagam, A. et al. Duration of infectiousness and correlation with RT-PCR cycle threshold values in cases of COVID-19, England, January to May 2020. *Euro Surveill* **25,** 2001483 (2020).
2. Quilty, B.J. et al. Quarantine and testing strategies in contact tracing for SARS-CoV-2: a modelling study. *Lancet Public Health* **6**, e175-e183 (2021).
3. Kissler, S.M. et al. SARS-CoV-2 viral dynamics in acute infections. *MedRxiv* doi: <https://doi.org/10.1101/2020.10.21.20217042> (2020).
4. University of Oxford. Preliminary report from the Joint PHE Porton Down & University of Oxford SARS-CoV-2 test development and validation cell: Rapid evaluation of Lateral Flow Viral Antigen detection devices (LFDs) for mass community testing (8 November 2020). <https://www.ox.ac.uk/sites/files/oxford/media_wysiwyg/UK%20evaluation_PHE%20Porton%20Down%20%20University%20of%20Oxford_final.pdf>
5. University of Liverpool. Liverpool Community Testing Pilot Interim Evaluation Report (23 December 2020). <https://www.liverpool.ac.uk/media/livacuk/coronavirus/Liverpool,Community,Testing,Pilot,Interim,Evaluation.pdf>
6. Guglielmi, G. Rapid coronavirus tests: a guide for the perplexed. *Nature* **590**, 203 – 205 (2021).
